# Structural Basis for Postfusion-Specific Binding to the Respiratory Syncytial Virus F Protein by the Canonical Antigenic Site I Antibody 131–2a

**DOI:** 10.1021/acsinfecdis.5c00368

**Published:** 2025-07-22

**Authors:** Weiwei Peng, Marta Šiborová, Xuesheng Wu, Wenjuan Du, Douwe Schulte, Matti F. Pronker, Cornelis A. M. de Haan, Joost Snijder

**Affiliations:** † Biomolecular Mass Spectrometry and Proteomics, Bijvoet Center for Biomolecular Research and Utrecht Institute of Pharmaceutical Sciences, 8125 Utrecht University, Padualaan 8, Utrecht 3584CH, the Netherlands; ‡ Virology Group, Division of Infectious Diseases and Immunology, Department of Biomolecular Health Sciences, Faculty of Veterinary Medicine, Utrecht University, Yalelaan 1, Utrecht 3584CL, the Netherlands

**Keywords:** respiratory syncytial virus, fusion protein, antibody sequencing, antigenic site I, 131−2a, epitope mapping

## Abstract

The respiratory syncytial virus (RSV) fusion (F) protein is a major target of antiviral antibodies following natural infection or vaccination and is responsible for mediating fusion between the viral envelope and the host membrane. The fusion process is driven by a large-scale conformational change in F, switching irreversibly from the metastable prefusion state to the stable postfusion conformation. Previous research has identified six distinct antigenic sites in RSV-F, termed sites Ø, I, II, III, IV, and V. Of these, only antigenic site I is fully specific to the postfusion conformation of F. A monoclonal antibody 131–2a that specifically targets postfusion F has been widely used as a research tool to probe for postfusion F and to define antigenic site I in serological studies, yet its sequence and precise epitope have remained unknown. Here, we use mass spectrometry-based *de novo* sequencing of 131–2a to reverse engineer a recombinant product and study the epitope to define antigenic site I with molecular detail, revealing the structural basis for the antibody’s specificity toward postfusion RSV-F.

## Graphical abstract

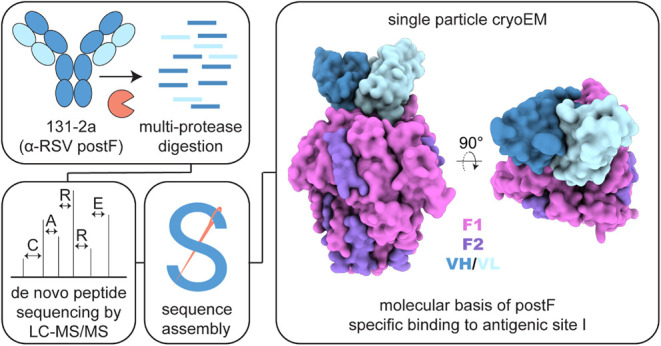

Respiratory syncytial virus (RSV) is the second major cause of hospital admissions for young infants globally, following malaria. It is estimated that RSV is responsible for approximately 60,000 childhood deaths each year, especially in low-resource settings.^1,2^ RSV is a member of the *Pneumoviridae* family. It consists of a negative-sense, single-stranded RNA genome enveloped in a lipid bilayer containing three virally encoded envelope proteins: the small hydrophobic protein (SHP), fusion protein (F), and glycoprotein (G).^3^ Both G and F are involved in host cell attachment and receptor binding, and as the name suggests, F also mediates cell entry by fusing the viral envelope with the host membrane, thereby delivering the genetic material of the virus into its host cell.^4^ The antibodies that target G and F play a pivotal role in the antiviral immune response, and F in particular has become the focus of vaccines and monoclonal antibody therapies that have been recently approved or are currently under clinical development.^5–7^

F is a trimeric class I viral fusion protein that exists in a metastable prefusion conformation. Its conformational change into a stable postfusion conformation drives fusion of the viral envelope with the host membrane. Owing to the drastic conformational changes between the pre- and postfusion states of F, each conformation presents distinct epitopes. Previous studies have described this complex antigenic landscape by defining six antigenic sites: Ø, I, II, III, IV, and V (see Figure 1).^11^ Antigenic sites Ø and V are specific to the prefusion state of F, and while antibodies directed against antigenic site III can bind F in both conformations, they also bind more strongly to the prefusion conformation. Antigenic sites II and IV are shared between pre- and postfusion states, but antibodies directed to antigenic site I are fully specific to the postfusion conformation.^8^

**Figure 1:**
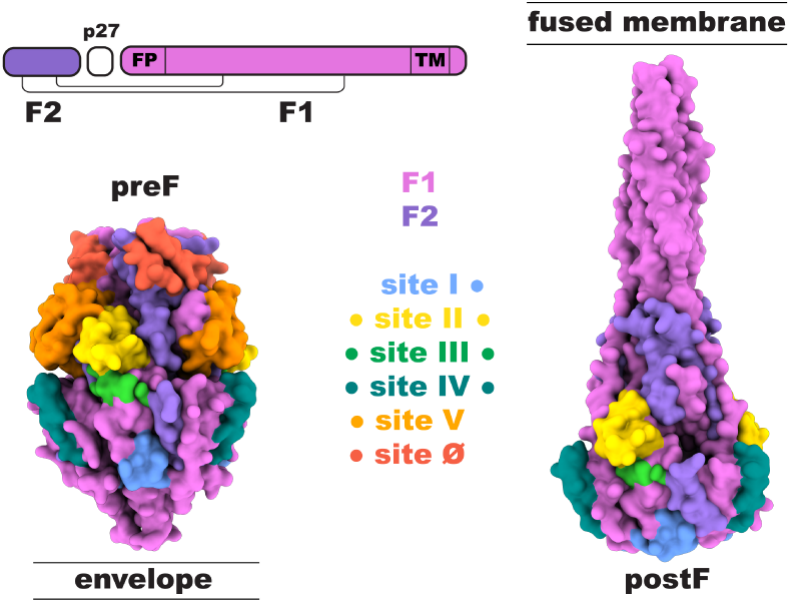
Antigenic sites on the RSV-F protein. The mature RSV-F antigen is proteolytically processed from the F0 precursor into disulfide-linked F1 and F2 subunits, releasing the p27 peptide. FP: fusion peptide, TM: transmembrane region. (left) Prefusion conformation of RSV-F (PDB ID: 4MMU).^8^ (right) Postfusion conformation of RSV-F (PDB ID: 3RRR).^9^ Antigenic sites are plotted on the surface with the indicated colors. Specificity of the antigenic sites for pre- and postF is indicated with the colored dots. Antigenic site definition follows Hause et al. 2017.^10^ The monoclonal antibody 131–2a is widely used to define antigenic site I.

Even in newly produced virions from an infected host cell, F is present on the envelope in a complex mixture of pre- and postfusion states. Meanwhile, neutralizing activity in immune sera can be largely attributed to prefusion F-specific antibodies. The postfusion F on virions has been speculated to act as a decoy to the immune system, directing it toward non-neutralizing epitopes at the expense of effectively neutralizing epitopes present in prefusion F. The antigenic site I-directed antibodies thereby constitute an important, possibly counterproductive component of the antibody response to natural RSV infection or vaccination.^12^

Serological studies have used monoclonal antibody standards to probe the antigenic site-specific antibody response in human subjects. This is typically done in competition binding experiments involving monoclonal antibody standards and polyclonal sera to pre- and postfusion F.^13,14^ The mouse monoclonal antibody 131–2a has become the canonical antigenic site I-defining antibody in these studies and is also widely used to specifically detect postfusion F in viral cultures and vaccine preparations by ELISA, Western blot, immunofluorescence microscopy, and cell sorting experiments. While 131–2a was discovered in the early 1980s and has since been widely used in RSV studies, its sequence is not publicly available.^15^ We have recently developed a mass spectrometry-based workflow to sequence antibodies straight from the purified protein,^16–18^ which we apply here to reverse engineer a functional recombinant 131–2a monoclonal antibody. Moreover, the epitope of 131–2a and other antigenic site I antibodies has historically been defined solely by the rise of escape mutants at P389 of the F1 subunit on postfusion F (or indirectly by competition with 131–2a).^11^ The molecular determinants of antigenic site I and its postfusion specificity have therefore remained elusive until finally in 2018, Goodwin and colleagues determined a crystal structure of the germline infant antibody ADI-14359 in complex with postfusion F.^19^ While ADI-14359 makes crucial contacts in the vicinity of P389, it has no direct physical interactions with the residue, which remains largely accessible. The structural basis of 131–2a and other antigenic site I antibodies’ specificity to postfusion F has therefore not been conclusively established. The reverse engineering of 131–2a described here enabled us to further detail the interaction of 131–2a with F by single-particle cryogenic electron microscopy (cryoEM), revealing the structural basis for its binding specificity to the postfusion conformation via a complementary mechanism to ADI-14359. These findings shed new light on decades of serological studies relying on 131–2a and provide a useful framework for understanding antigenic site I antibodies and their specificity toward postfusion F.

## Results

### *De Novo* Sequencing 131–2a by Mass Spectrometry-Based Bottom-Up Proteomics

The purified mouse monoclonal antibody 131–2a was sequenced by mass spectrometry using a bottom-up proteomics approach. The antibody was digested with a panel of 7 proteases in parallel (trypsin, chymotrypsin, α-lytic protease, thermolysin, elastase, lysC, and lysN) to generate overlapping peptides for the LC–MS/MS analysis, using an in-solution digestion protocol. Peptides were sequenced from MS/MS spectra, following a hybrid fragmentation scheme with both stepped high-energy collision dissociation (sHCD) and electron-transfer high-energy collision dissociation (EThcD) on all peptide precursors. The peptide sequences were predicted from the MS/MS spectra using PEAKS and assembled into the full-length heavy and light chain sequences using the in-house-developed software Stitch.

This resulted in the identification of a mouse IgG2a antibody with an IGHV1S29 heavy chain, paired with an IGKV3-2 light chain (the full sequence is provided in the Supporting Information). The depth of coverage for the complementarity determining regions (CDRs) varies from around 10 to 200, indicating high sequence accuracy (see Supplementary Figure S1). Examples of MS2 spectra supporting the CDRs of both the heavy chain and light chain are shown in Figure 2. The heavy and light chains exhibit a typical moderate degree of somatic hypermutation, estimated at 12% and 5%, respectively. This includes several inferred mutations in the framework regions of the chains.

**Figure 2:**
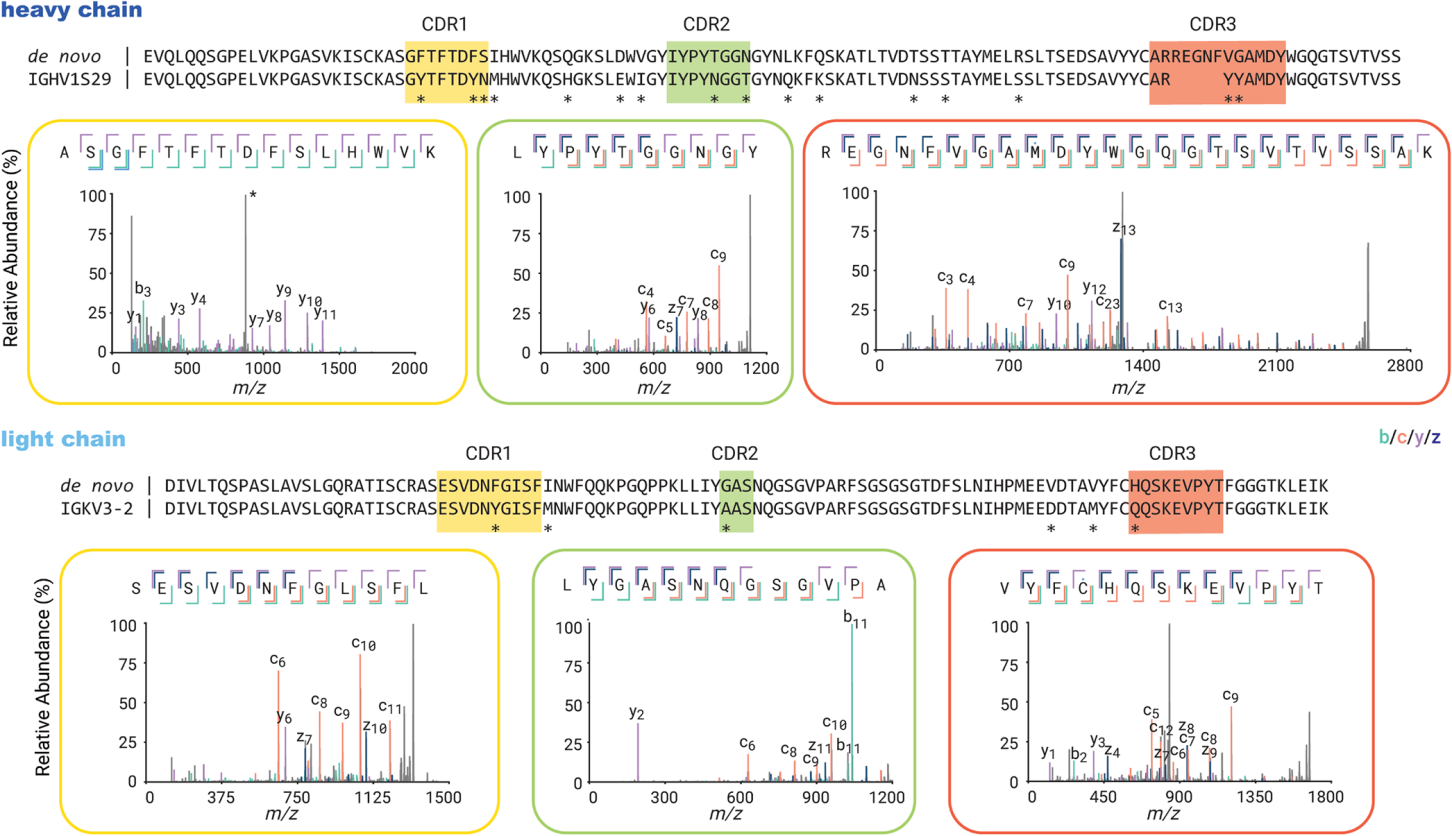
*De novo* sequencing of 131–2a by mass spectrometry-based bottom-up proteomics. The variable region alignment to the inferred germline sequence is shown for both heavy and light chains. Positions with putative somatic hypermutation are highlighted with asterisks (*). The MS2 spectra supporting the complementarity determining regions (CDRs) are shown beneath the sequence alignment.

### Reverse Engineering a Functional 131–2a Monoclonal Antibody

The experimentally determined sequences of 131–2a were reverse translated to DNA with codon optimization for expression in HEK293E cells. The synthetic DNA for the variable domains was inserted into the mouse IgG2a backbone with a C-terminal His_8_-tag on the heavy chain for purification and the mouse Ig Kappa backbone for the light chain. Plasmids for the heavy and light chains were cotransfected into HEK293E cells, with the recombinant 131–2a yielding 95 mg from a 1 L culture after His-tag purification. The reverse-engineered 131–2a was then compared with the input material for sequencing using Western blot and enzyme-linked immunosorbent assay (ELISA). As shown in Figure 3, the reverse-engineered 131–2a binds specifically to postF in a manner that is indistinguishable from that of the input material. This demonstrates that the mass spectrometry-derived sequence yielded a functionally equivalent antibody product.

**Figure 3:**
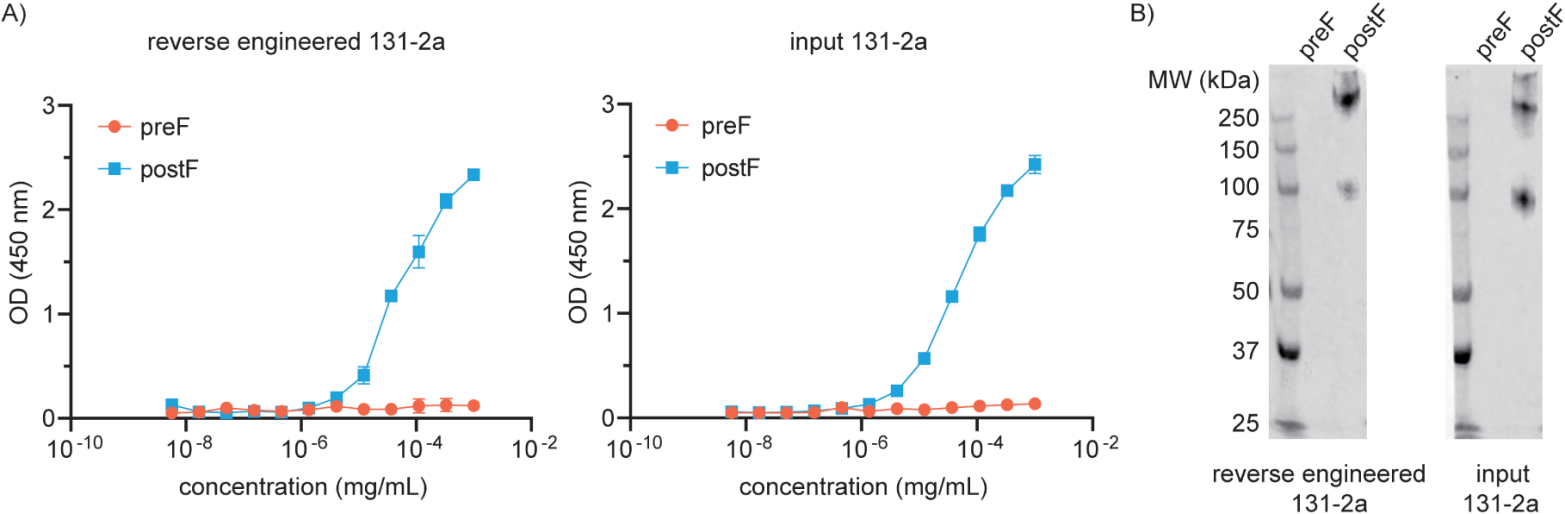
Validation of recombinant 131–2a expressed with a mass spectrometry-derived sequence. (A) ELISA of pre/postF comparing the sequenced input and reverse-engineered 131–2a. (B) Western blot analysis following nondenaturing PAGE of pre/postF comparing the sequenced input and reverse-engineered 131–2a. The bands at 100 kDa and >250 kDa represent F monomers and trimers, respectively.

### Epitope Mapping of 131–2a

Despite its use as an antibody standard to define antigenic site I and specifically detect the F protein in a postfusion state, the molecular basis of 131–2a’s postfusion specificity is not well understood. Reverse engineering 131–2a enabled us to map the epitope in greater detail using single-particle cryoEM (Supplementary Figure S2). Our reconstructions of the F:131–2a complex recovered a clearly identifiable postF head domain, while the stalk remained largely unresolved. The imaged particles consist of a mixture of three F:131–2a stoichiometries, containing 3:0, 3:1, and 3:2 subunits of each component. The 3:1 complex was the most populated in this dataset and refined to a resolution of 3.2 Å. The final map at 3.1 Å resolution reported here is based on C3 reconstructions with symmetry relaxation of the 3:1 and 3:2 complexes, followed by symmetry expansion and combining all poses with a bound Fab into a final local refinement with the constant domains of the Fabs masked out (Supplementary Table S1).

The epitope of 131–2a spans a single protomer of the postF trimer, burying 1308 Å^2^ of its accessible surface area. Interactions are mediated by all six CDRs of both the heavy- and light-chain variable domains (see Figure 4). The epitope is a composite of three discontinuous regions from the F1 and F2 subunits, spanning residues 31–42 (F2), 323–332 (F1), and 379–399 (F1). Full lists of buried residues and hydrogen bonds are available in Supplementary Tables S2 and S3.

**Figure 4:**
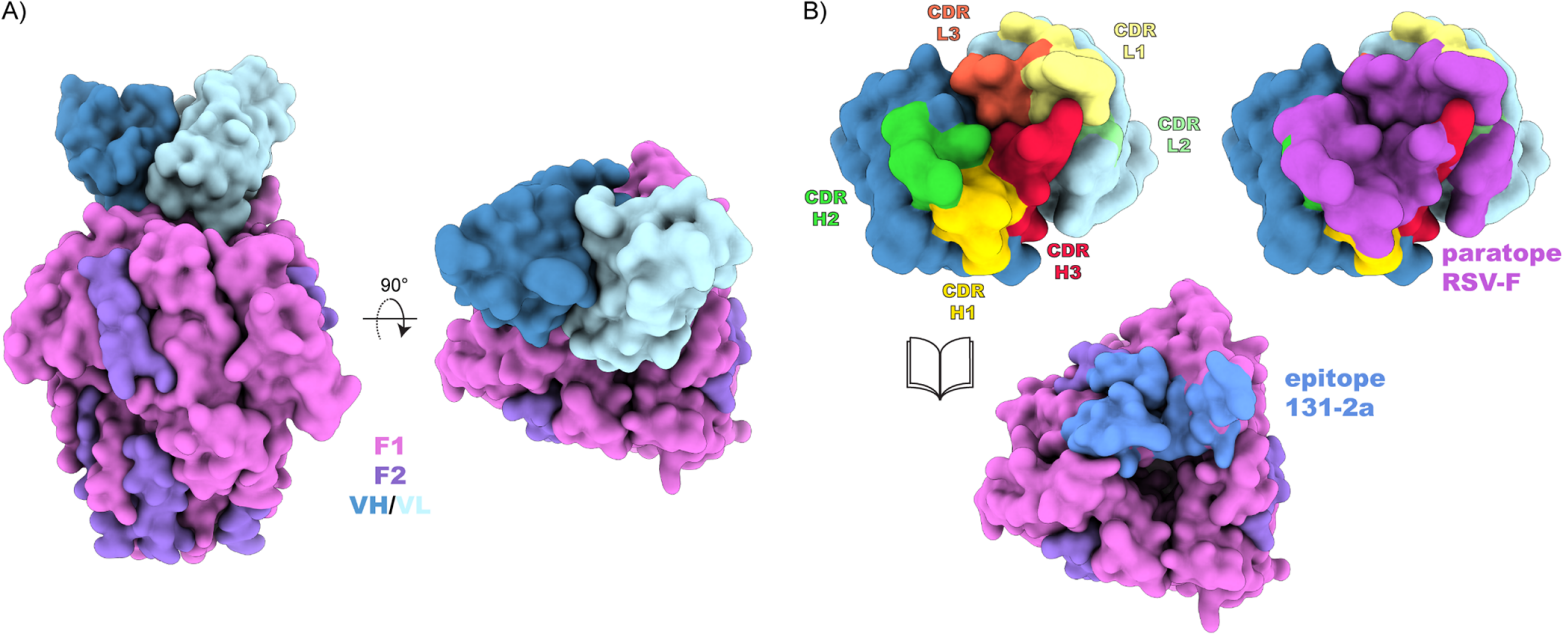
Atomic model of RSV postF bound to the 131–2a Fab. (A) Surface representation of postF bound to a single copy of 131–2a’s VH/VL domains. (B) Detailed view of the buried surface between 131–2a (paratope) and postF (epitope).

The most extensive contacts are made within residues 379–399 of the F1 subunit (see Figure 5A), a looped region exposed on the surface of the postF apex, facing the internal cavity of the trimer’s head domain and stabilized by an internal disulfide bridge (C383–C393). It includes P389, which is a site of known escape mutants to 131–2a and historically used to define the epitope at antigenic site I for this and other monoclonal antibodies.^9,11^ The peptide bond between P389 and K390 makes a hydrogen bond with the Y55 side chain in the heavy chain framework region 2 (directly flanking CDRH2). The same Y55 residue of the heavy chain is also hydrogen bonded to the side chain of F1–K390, which, in turn, is also hydrogen bonded to the backbone of N65 in CDRH2. Residue F1–D385 appears to be another critical part of the epitope, forming hydrogen bonds with both the Y57 side chain of CDRH2 and the backbone of N110 in CDRH3. While the light chain also makes extensive contacts with the 379–399 region, these appear to be exclusively van der Waals interactions.

**Figure 5:**
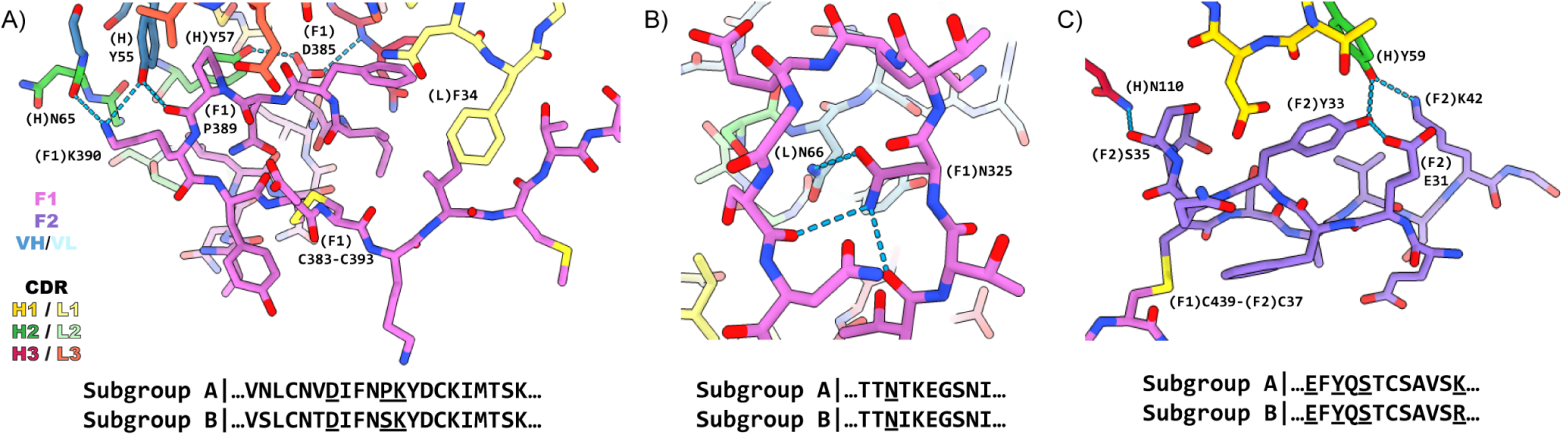
Detailed interactions in the 131–2a epitope on postF. (A) Interactions with residues 379–399 of the F1 subunit. (B) Interactions with residues 323–332 (C322–C333 loop) in the F1 subunit. (C) Interactions with residues 31–42 of the F2 subunit. Key residue numbers with associated chains are indicated, and hydrogen bonds are indicated with dashed blue lines. The RSV-F sequence is highly conserved within subgroups A and B, with the corresponding consensus sequences of the epitope displayed. Hydrogen-bonded residues to 131–2a are underlined.

Additional contacts are made by the light chain with the loop between the C322–C333 disulfide bridge in the F1 subunits (Figure 5B). This loop is disordered in previously published crystal structures of postF,^9^ as well as in the unbound protomers in the map/model reported here. The loop is stabilized in the interaction with 131–2a by a hydrogen bond between F1–N325 and residue N66 in the framework region 3 of the light chain (directly flanking CDRL2). The 131–2a epitope is completed by interactions of the heavy chain with residues 31–42 of the F2 subunit at the apex of the postF head domain (Figure 5C). This region spans the disulfide bridge between the F1 and F2 subunits (at F1–C439 and F2–C37). The interaction includes hydrogen bonds between the backbone of F2–S35 and the N110 side chain of CDRH3, as well as between F2–Y33 and F2–K42 with Y59 of CDRH2.

The determined model of the 131–2a interaction with postF can be used to rationalize most of the somatic hypermutations observed in the mature VH/VL sequences compared to the inferred germline precursors (Supplementary Figure S3). The mutated sites within or directly flanking the CDRs of both the heavy and light chains are directly involved in the interaction with postF. In addition, we inferred mutations in both the heavy and light chains that are directly situated at the interface of the VH/VL domains, possibly involved in the pairing of the chains. Finally, the IGHV1S29 germline of the heavy chain carries a predicted N-linked glycosylation site at N82 of the framework 3 region. A putative glycan at this location is predicted to shield a large portion of the paratope, rationalizing the observed N82T mutation in the mature 131–2a sequence to allow free access to the postF interface in the absence of the N-linked glycan (Supplementary Figure S3B).

Based on the epitope determined here, the structural basis for 131–2a’s specificity to the postF conformation can be explained (see Figure 6). While the conformation of the epitope itself remains stable between preF and postF (Supplementary Figure S4), access to the F1–379–399 region is sterically blocked by the C-terminus of the F1 subunit in the preF state. This region contains the α10 helix of the heptad repeat B (HRB) region, which refolds to the helical stalk domain of the postF state. The specificity of 131–2a to postF is therefore negatively determined by steric blocking of the epitope in the preF conformation. This mechanism is broadly similar to the postfusion specificity of another site I-directed antibody, ADI-14359,^19^ though it differs in some crucial details of how the epitope is blocked in the preF state (see Figure 7). While 131–2a binds at the apex of the postF head domain at a rather sharp angle relative to the symmetry axis of the trimer, the angle of approach is more obtuse and the epitope more peripheral for ADI-14359. Contacts by ADI-14359 are mediated for the largest part by the heavy chain, but despite the minor contributions of the light chain to binding postF, it plays a critical role in mediating the postF specificity. The epitope of ADI-14359 is still fully accessible in the preF conformation and is not blocked by the α10 helix of the F1–HRB region. Rather, the extended C-terminus of the F1 subunit, directly preceding the α10 helix, augments the antiparallel β-sheet formed by the looped 31–42 region of F2, which is shared in both 131–2a and ADI–14359 epitopes and produces a steric clash with the ADI–14359 light chain in the preF conformation.

**Figure 6:**
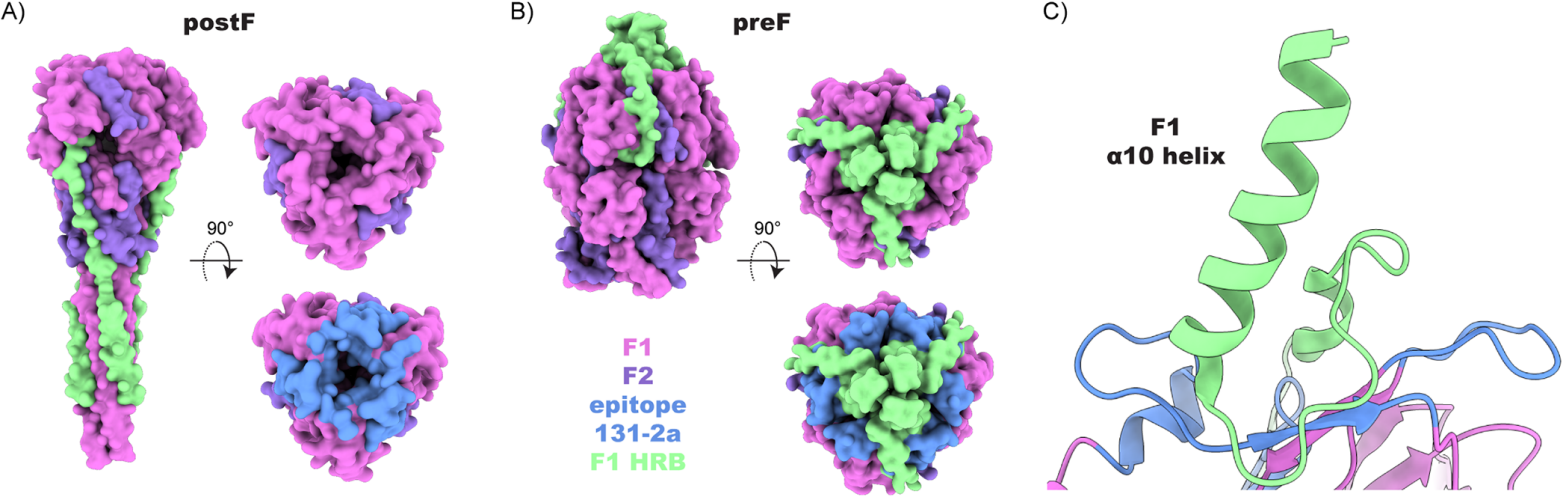
Structural basis for postF specificity of 131–2a. (A) Surface representation of the postF crystal structure (PDB ID: 3RRR). (B) Surface representation of the preF crystal structure (PDB ID: 4MMU). (C) Cartoon representation of a single preF protomer at the 131–2a epitope. While the conformation of the 131–2a epitope remains the same in preF versus postF, access to the epitope is blocked in preF by the α10 helix and heptad repeat B (HRB) of the F1 C-terminus, which refolds into the helical stalk region of postF.

**Figure 7:**
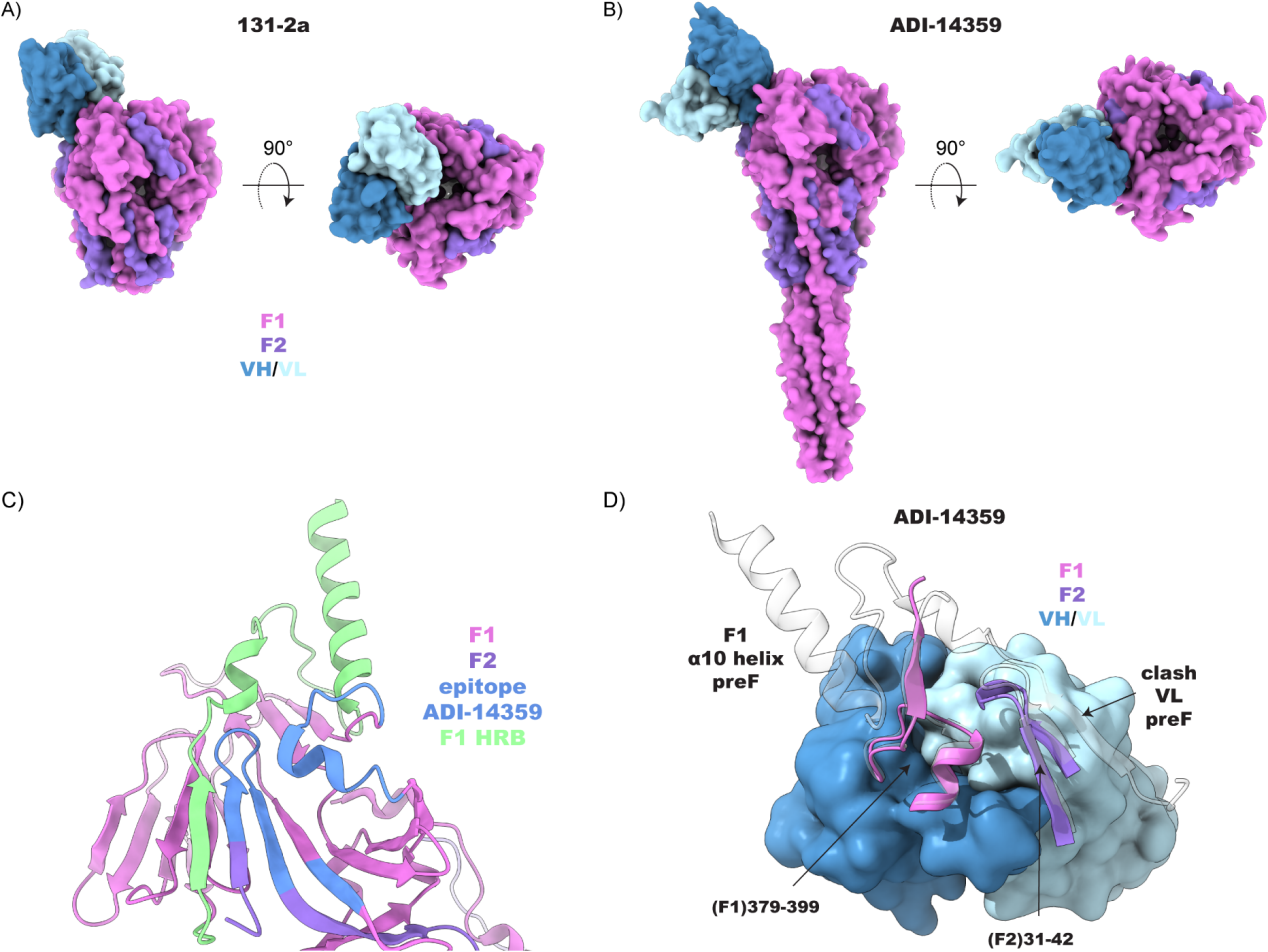
Comparison of the 131–2a epitope (A) with the antigenic site I antibody ADI–14359 (PDB ID: 6APB) (B). The cartoon in (C) shows the ADI–14359 epitope mapped onto a single protomer in preF (PDB ID: 4MMU). While the ADI–14359 epitope remains exposed, the extended C-terminus of F1 augments the β-sheet of the F2 region, sterically clashing with the light chain of ADI–14359 (D).

Curiously, the structure of postF bound to ADI–14359, like 131–2a, was also solved in a 3:1 stoichiometry. While we cannot exclude that, in both cases, the substoichiometric binding is a sample preparation or processing artifact, it is interesting to note that the 131–2a epitope determined here would predict a very close fit for the fully occupied 3:3 complex (see Supplementary Figure S5). The light chains of the three 131–2a variable domains point inward over the central cavity of the postF trimer, and while technically no direct clashes are observed, there is no space for as much as a water molecule between them. This may result in less favorable binding energies for a second and third bound Fab and could go some way to explain the low occupancy observed in our preparations, despite the molar excess of 131–2a Fab added to postF.

## Conclusions

In this work, the application of mass spectrometry-based bottom-up proteomics allowed us to derive the full sequence of the canonical antigenic site I-defining, anti-RSV-F monoclonal antibody 131–2a. This enabled the reverse engineering of a functional recombinant 131–2a antibody, which was demonstrated to possess binding specificity equivalent to that of RSV postF when compared to the sequenced input material.

This in turn enabled a single-particle cryoEM of 131–2a in complex with postF to map its epitope in detail. It revealed a composite epitope encompassing residues 379–399 in the F1 subunit, with additional stabilizing contacts to the loop spanning C322–C333 in F1, and extensive contacts with residues 31–42 in F2. While the conformation of the epitope remains similar in the prefusion conformation of F, access is hindered by the C-terminus of the F1 subunit, which refolds to the stalk region in the postfusion conformation. The postF specificity of 131–2a can thus be explained by the negative selection of binding in the prefusion conformation. *De novo* sequencing of 131–2a by mass spectrometry enabled an in-depth molecular characterization of antigenic site I of the RSV-F protein, shedding new light on decades of serological studies characterizing the antibody response to RSV infection and vaccination.

## Methods

### Expression and Purification of RSV-F Proteins

Design, expression, and purification of RSV prefusion F (DSCav1-T4fd, Genbank JX015498) have been described previously.^14,20^ Briefly, cDNA encoding prefusion RSV F (DSCav1-T4fd) was cloned into the pCD5 expression vector in frame with the CD5 signal peptide coding sequence, followed by sequences encoding a C-terminal T4 fibritin trimerization motif, thrombin site, and StrepII-tag (IBA, Germany). Prefusion F was expressed transiently in HEK-293T cells [ATCC, CRL-11268] and the secreted protein was purified from culture supernatants using Strep-tactin Sepharose beads (IBA) following the manufacturer’s protocol and as described previously.^14,21^ RSV postfusion F corresponds to Flys-GCN described previously, except that the StrepII-tag was not present in the current protein and that it was expressed in CHO cells.^21^ This construct was generated by cloning cDNAs encoding the RSV F ectodomain (amino acids 26 to 515, GenBank: JX015498.1) in frame with a CD5 signal peptide-encoding sequence, followed by sequences coding for the GCN4 isoleucine zipper trimerization motif and a LysM peptidoglycan binding domain.^22,23^ In addition, arginines in the two furin-cleavage sites were substituted by lysines. This protein was previously used in a clinical trial^24^ and did not display D25 reactivity after its prolonged storage at −80 °C.

### MS-Based Sequencing of 131–2a

#### Sample Preparation

24 μg of 131–2a (MAB8599P–K, Sigma) was denatured and reduced in 2% sodium deoxycholate (SDC), 200 mM Tris-HCl, 10 mM tris­(2-carboxyethyl)­phosphine (TCEP), and 40 mM iodoacetic acid, pH 8.5 at 95 °C for 10 min, followed by 20 min incubation at room temperature in the dark for alkylation. 3 μg of sample was then digested by one of the following proteases, trypsin, chymotrypsin, α-lytic protease, thermolysin, elastase, lysC, and lysN in a 1:50 ratio (w:w) in a total volume of 100 μL of 50 mM ammonium bicarbonate at 37 °C overnight. After digestion, SDC was removed by adding 2 μL of formic acid (FA) and centrifuging at 14000*g* for 20 min. Following centrifugation, the supernatant containing the peptides was collected for desalting on a 30 μm Oasis HLB 96-well plate (Waters). The Oasis HLB sorbent was activated with 100% acetonitrile and subsequently equilibrated with 10% formic acid in water. Next, peptides were bound to the sorbent, washed twice with 10% formic acid in water, and eluted with 100 μL of 50% acetonitrile/5% formic acid in water (v/v). The eluted peptides were vacuum-dried and reconstituted in 100 μL of 2% FA.

#### Mass Spectrometry

The digested peptides (single injection of 0.2 μg) were separated by online reversed-phase chromatography on an Agilent 1290 UHPLC (column packed with Poroshell 120 EC C18; dimensions 50 cm × 75 μm, 2.7 μm, Agilent Technologies) coupled to a Thermo Scientific Orbitrap Fusion mass spectrometer. Samples were eluted over a 90 min gradient from 0% to 35% acetonitrile at a flow rate of 0.3 μL/min. Peptides were analyzed with a resolution setting of 60000 in MS1. MS1 scans were obtained with a standard AGC target, a maximum injection time of 50 ms, and a scan range of 350–2000. The precursors were selected with a 3 *m*/*z* window and fragmented by stepped HCD as well as EThcD. The stepped HCD fragmentation included steps of 25%, 35%, and 50% NCE. EThcD fragmentation was performed with calibrated charge-dependent ETD parameters and 27% NCE supplemental activation. For both fragmentation types, MS2 scans were acquired at 30000 resolution, 800% normalized AGC target, 250 ms maximum injection time, and a scan range of 120–3500.

#### Data Analysis

MS/MS spectra were used to determine *de novo* peptide sequences using PEAKS Studio (version 11.5).^25^ We used tolerances of 20 ppm and 0.02 Da for MS1 and MS2, respectively. Carboxymethylation was set as a fixed modification of cysteine. Oxidation of methionine, tryptophan, and histidine and pyroglutamic acid modification of *N*-terminal glutamic acid and glutamine were set as additional variable modifications. The CSV file containing all the *de novo* sequenced peptides was exported for further analysis. Stitch (version 1.5) was used for the template-based assembly.^26,27^ The mouse antibody database from IMGT was used as templates.^28,29^ The cutoff score for the *de novo* sequenced peptide was set at 85, and the cutoff score for the template matching was set at 16. A first iteration was performed with automatic V–J segment joining, which failed for the heavy chain due to minimal overlap between the overhanging reads. The CDRH3 region was constructed manually from the overhanging peptides assembled at the ends of the V and J segments, followed by a second iteration of Stitch, assembling the input reads to the first iteration draft sequences of the full heavy and light chains. The resulting consensus sequence was manually corrected to include the conserved cysteine residues at the start of CDRH1 and CDRH3. The determined variable domain sequences were plugged into the identified constant domain sequences from Uniprot to arrive at the final sequences reported here. V-genes and CDR boundaries were assigned with the IMGT DomainGapAlign Web server (https://www.imgt.org/3Dstructure-DB/cgi/DomainGapAlign.cgi).

### Cloning and Expression of Recombinant 131–2a

To recombinantly express full-length 131–2a, the proteomic sequences of both the light and heavy chains were reverse-translated and codon-optimized for expression in human cells using the Thermo Fisher webtool (https://www.thermofisher.com/order/gene-design/index.html). For the linker and Fc region of the heavy chain, the standard mouse IgG2A amino acid sequence (IMGT database) was used. An N-terminal secretion signal peptide derived from the human IgG light chain (MEAPAQLLFLLLLWLPDTTG) was added to the N-termini of both the heavy and light chains. *Bam*HI and NotI restriction sites were added to the 5′ and 3′ ends of the coding regions, respectively. Only for the light chain, a double stop codon was introduced at the 3′ site before the NotI restriction site. The coding regions were subcloned using *Bam*HI and NotI restriction-ligation into a pRK5 expression vector with a C-terminal octahistidine tag between the NotI site and a double stop codon 3′ of the insert, so that only the heavy chain has a C-terminal AAAHHHHHHHH sequence for nickel-affinity purification (the triple alanine resulting from the NotI site). After the sequence was validated by Sanger Sequencing, the HC/LC was mixed in a 1:1 DNA ratio and expressed in HEK293E cells by the ImmunoPrecise Antibodies (Europe) B.V. company. After expression, the culture supernatant of the cells was harvested and purified using HisPur Ni-NTA Resin (Thermo Fisher Scientific). After purification, 131–2a was buffer-exchanged and concentrated into phosphate-buffered saline (PBS) by using a centrifugal filter (Amicon). The expression plasmids described above are available through Addgene (https://www.addgene.org/Joost_Snijder/).

### 131–2a Fab Generation

The full 131–2a IgG was digested by immobilized papain (Thermo Fisher) in digestion buffer (0.22 mM cysteine HCl, 20 mM phosphate buffer, pH 7) at 37 °C with 1000 rpm shaking for 5 h. After digestion, the Fc segment was removed by incubation with protein A agarose resin (Thermo Fisher Scientific) at room temperature for 15 min. The 131–2a Fab was further purified by size-exclusion chromatography using a Superdex 200 Increase 10/300 GL column (Cytiva) equilibrated in PBS buffer.

### Western Blot

Binding of 131–2a was analyzed by Western blot assay utilizing both prefusion and postfusion proteins mentioned above.^14,20,21^ Briefly, 0.25 μg of either post- or prefusion RSV F proteins was mixed with native protein buffer (Bio-Rad, 1610738) and then loaded onto a 7% polyacrylamide gel devoid of SDS, accompanied by protein standards (Bio-Rad, 1610375). The proteins were transferred to a cellulose nitrate membrane using a Trans-Blot Turbo Transfer System (Bio-Rad). Following this, the membrane was subjected to blocking using 3% BSA alongside 0.1% Tween 20. Horseradish peroxidase (HRP)-conjugated rabbit antimouse IgG (Dako, P0260) was used at a dilution of 1:5,000 for detection of the 131–2a antibody. Visualization was performed by using BeyoECL Moon (Beyotime). The Western blots were scanned using an imaging system (Odyssey).

### ELISA

Nunc MaxiSorp ELISA plates (Thermo Fisher Scientific) were coated with 50 ng of RSV F and incubated overnight at 4 °C, followed by three washing steps with PBS containing 0.05% Tween 20. Plates were blocked with 2% bovine serum albumin (BSA; Fitzgerald) in PBS with 0.1% Tween 20 at 4 °C overnight. Subsequently, 131–2a (homemade or commercial (MAB8599P-K, Sigma)) antibodies were allowed to bind the plates at 3-fold serial dilutions, starting at 1 μg/mL diluted in PBS containing 2% BSA and 0.1% Tween 20, at RT for 1 h. After washing, plates were incubated with 1:1000 diluted HRP-conjugated rabbit antimouse IgG (Dako, P0260) for 1 h at RT. HRP reactivity with a tetramethylbenzidine substrate (BioFX) was determined by measuring absorption at 450 nm using an ELISA plate reader (EL-808, BioTek).

### CryoEM Sample Preparation

RSV postF and 131–2a Fabs were mixed in a 3:4 molar ratio and incubated for 30 min on ice. The sample was diluted to 0.2 mg/mL in PBS and was pipetted onto a holey carbon-coated copper grid (R1.2/1.3, mesh 200; Quantifoil), blotted, and vitrified by plunging into liquid ethane using an FEI Vitrobot Mark IV (Thermo Fisher Scientific). Prior to sample deposition, grids were glow-discharged for 60 s at 10 mA. A sample volume of 4 μL was applied, using a blot force of 0, with blotting and waiting times of 0 and 5 s, respectively.

### CryoEM Data Collection

The vitrified sample was transferred to a Titan Krios electron microscope (Thermo Fisher Scientific) operated under cryogenic conditions and at an acceleration voltage of 300 kV. The Gatan BioQuantum energy filter was operated with a slit width of 20 eV. The C1, C2, and objective apertures were 2000, 50, and 100 μm, respectively. A total of 4330 micrographs were collected at a magnification of 105,000× on a K3 direct electron detector in counted super-resolution mode at a calibrated pixel size of 0.418 Å/pix, binned to a final pixel size of 0.83 Å/pix. Imaging was done under low-dose conditions (total dose 50 e^–^/Å^2^ and defocus values ranging from −0.8 to −2.0 μm. The 2.52 s exposure was fractionated into 50 frames and saved in TIFF format. Automated data acquisitions were performed by using the software EPU with AFIS (Thermo Fisher Scientific).

### CryoEM Data Processing

Data was processed in Cryosparc using successive versions from v4.2.1 to v4.5.3.^30^ The movies were motion-corrected and 2x binned using PatchMotionCorrection. CTF parameters were estimated with the PatchCTF function using default parameters. Micrographs were filtered to an estimated maximum resolution from PatchCTF of <5 Å, selecting 3802 for the final processed dataset.

An initial set of 2.1 M particles was picked with the Blobpicker function, extracted using an original box size of 360 px and binned 4x for further processing. Following two successive rounds of 2D classification, 563k particles were selected for Ab Initio reconstruction into 8 classes. The resulting volumes could be grouped as RSV-F trimers with 0, 1, or 2 copies of the bound Fab, along with a smaller unidentified class, somewhat resembling a free Fab. Particles corresponding to postF trimers with either 0, 1, or 2 Fabs were re-extracted with a 360 px box size binned 1.5×, resulting in maps of 3.4, 3.3, and 3.4 Å resolution, respectively, following nonuniform refinement.

The postF + 1 Fab map was used to generate templates for the template picker function, resulting in 2.4 million new picks. Using the previously reconstructed maps of postF + 0, +1 or +2 Fabs as starting volumes, along with the ab initio class of the smaller Fab-like class, the full stack of 2.4 M particles (original box size 360 px binned 4×) was used as input for an initial round of heterorefinement. The particles for each assigned volume were then subjected to two additional successive rounds of heterorefinement with the same 4 starting volumes, selecting only the particles for the target class for each new round. This resulted in 186k particles classified as postF + 0 Fab, 200k particles +1 Fab, and 97k particles +2 Fabs.

After extraction of the classified particles with the original box size of 360px binned 1.5×, the particles were reconstructed to 3.1, 3.2, and 3.3 Å resolution for +0, +1, +2 Fabs, respectively, following successive nonuniform and local refinements (C1 symmetry). The maps for postF with +1 and +2 Fabs were then reconstructed with C3 symmetry using symmetry relaxation (marginalization), the maps aligned, the symmetry expanded, and the poses with Fabs bound combined into the final reconstruction at 3.1 Å resolution following local refinement with the constant domains of the Fab masked out.

### CryoEM Model Building and Refinement

Software used in this project was curated by SBGrid.^31^ As a starting point for the postF portion of the model, a homology model of our RSV-F construct based on the published postF crystal structure (PDB ID: 3RRR^9^) prepared using the SWISS-MODEL Web server, was rigid-body fitted using the “fit in map” function of ChimeraX (v1.8).^32,33^ The VH/VL structure of 131–2a was predicted with the SAbPred ABodyBuilder2 webserver, adopting IMGT numberings for the chains.^34^ The predicted VH/VL structure was rigid-body fitted in ChimeraX and combined into the same model with the postF trimer. Density for the loop spanning the C322–C333 disulfide bridge of the F1 subunit was only visible in the 131–2a-bound protomer of the postF trimer, which was manually fitted using Coot (WinCoot v0.9.8.1).^35^ The resulting model was used as input for flexible fitting using the Namdinator Web server with default parameters.^36^ Regions outside the map (termini, C322–C333 loops of the two unbound postF protomers, and the HRA/HRB helical stalk region) were pruned from the model, followed by a final round of flexible fitting in Namdinator with default settings. One final round of refinement in Phenix (v1.21.2.5419) using a nonbonded weight of 500 resulted in the final model reported here.^37^ Interfaces were analyzed using the PISA webserver.^38^ All figures were prepared using ChimeraX (v1.8).

The model of glycosylated 131–2a T82N was prepared by structure prediction with the SAbPred ABodyBuilder2 webserver, followed by the grafting of an A1F glycan with the GLYCOSHIELD webserver.^39^

## Supplementary Material



## Data Availability

The raw LC–MS/MS files and analyses have been deposited to the ProteomeXchange Consortium via the PRIDE partner repository with the dataset identifier PXD059427. The final reported map has been deposited in EMDB under ID 52444. The additional maps of RSV-F with 0, 1, and 2 Fabs bound are deposited in EMDB under IDs 52446, 52447, and 52448, respectively. The atomic model of postF with bound VH/VL of 131–2a has been deposited to the Protein Data Bank with the accession code 9HVW. The plasmids for 131–2a expression in mammalian cells are made available through Addgene under plasmid IDs 214965 and 215331 for the heavy and light chains, respectively.

## Notes

Full-length 131–2a heavy and light chain sequences in FASTA format, additional experimental details for the cryoEM reconstructions, tables of epitope-paratope contact residues, depth of coverage profiles for the experimental 131–2a sequence, and additional structural analyses of 131–2a binding (PDF)
