# Supplementary material for: Structural Basis for Postfusion-Specific Binding to the Respiratory Syncytial Virus F Protein by the Canonical Antigenic Site I Antibody 131–2a
Source: ACS Infect Dis. 2025 Jul 22;11(8):2357–66. doi: 10.1021/acsinfecdis.5c00368 (PMC12340942; doi:10.1021/acsinfecdis.5c00368)
Supplement: Supplementary file 1 [file id5c00368_si_001.pdf]

Supplementary information:

Structural basis for postfusion-specific binding to Respiratory Syncytial Virus F protein by the canonical antigenic site I antibody 131-2a.

Weiwei Peng[1a], Marta Šiborová[1a], Xuesheng Wu[2], Wenjuan Du[2], Douwe Schulte[1], Matti F. Pronker[1], Cornelis A. M. de Haan[2], Joost Snijder[1]*

[1] Biomolecular Mass Spectrometry and Proteomics, Bijvoet Center for Biomolecular Research and Utrecht Institute of Pharmaceutical Sciences, Utrecht University, Padualaan 8, 3584CH Utrecht, The Netherlands

[2] Virology Group, Division of Infectious Diseases and Immunology, Department of Biomolecular Health Sciences, Faculty of Veterinary Medicine, Utrecht University, Utrecht, Yalelaan 1, 3584CL, the Netherlands

[a] equal contribution

* corresponding author: j.snijder@uu.nl

Supplementary Information:

>131-2a_heavychain
EVQLQQSGPELVKPGASVKISCKASGFTFTDFSIHWVKQSQGKSLDWVGYIYPYTGGNGYNLK
FQSKATLTVDTSSTTAYMELRSLTSEDSAVYYCARREGNFVGAMDYWGQGTSVTVSSAKTTA
PSVYPLAPVCGDTTGSSVTLGCLVKGYFPEPVTLTWNSGSLSSGVHTFPAVLQSDLYTLSSSV
TVTSSTWPSQSITCNVAHPASSTKVDKKIEPRGPTIKPCPPCKCPAPNLLGGPSVFIFPPKIKDV
LMISLSPIVTCVVVDVSEDDPDVQISWFVNNVEVHTAQTQTHREDYNSTLRVVSALPIQHQDW
MSGKEFKCKVNNKDLPAPIERTISKPKGSVRAPQVYVLPPPEEEMTKKQVTLTCMVTDFMPEDI
YVEWTNNGKTELNYKNTEPVLDSDGSYFMYSKLRVEKKNWVERNSYSCSVVHEGLHNHHTTK
SFSRTPGK

>131-2a_lightchain
DIVLTQSPASLAVSLGQRATISCRASESVDNFGISFINWFQQKPGQPPKLLIYGASNQGSGVPA
RFSGSGSGTDFSLNIHPMEEVDTAVYFCHQSKEVPYTFGGGTKLEIKRADAAPTVSIFPPSSEQ
LTSGGASVVCFLNNFYPKDINVKWKIDGSERQNGVLNSWTDQDSKDSTYSMSSTLTLTKDEYE
RHNSYTCEATHKTSTSPIVKSFNRNCCSNTTGSKT

Supplementary Table S1. CryoEM data collection and refinement statistics.

**Reconstruction**	**RSV-F postfusion trimer with one 131-2a Fab**
**Data collection and processing**	**EMDB-52444/52446/52447/52448**
Magnification	105000
Voltage (kV)	300
Electron exposure (e-/Å^2)	50
Defocus range (μm)	-0.8 to -2.0
Pixel size (Å)	0.83
Symmetry imposed	C1/C3/C1/C1
Map resolution (Å)	3.1/3.1/3.2/3.3
FSC threshold	0.143
**Refinement**	**PDB ID: 9HVW**
Initial model used (PDB ID)	3RRR
Map sharpening *B* factor (Å^2)	-105
# non-hydrogen atoms	8961
# residues	1149
B factor (Å^2)	-84
R.m.s. deviations	
Bond lengths (Å)	0.004
Bond angles (°)	0.817
Validation	
MolProbity score	1.8
Clashscore	6.17
Poor rotamers (%)	1.53
Ramachandran plot	
Favored (%)	95.37
Allowed (%)	4.63
Disallowed (%)	0

Supplementary Table S2. Buried residues in the 131-2a interaction with postF (from PISA).

**RSV-F_F1 (chain E)**	**RSV-F_F2 (chain B)**	**131-2a HC (chain H)**	**131-2a LC (chain L)**
321L	31E	29T	31N
323T	32F	35T	34F
325N	33Y	36D	35G
327K	34Q	37F	36I
328E	35S	38S	38F
330S	36T	40H	55Y
332I	40V	52W	65S
334L	42K	55Y	66N
336R		57Y	67Q
377S		59Y	68G
378E		62T	69S
380N		64G	70G
381L		65N	107S
383N		66G	108K
384I		107R	109E
385D		108E	114V
386I		109G	116Y
387F		110N	
388N		112V	
389P			
390K			
392D			
395I			
396M			
397T			
399K			

contact
H-bonded residue

Supplementary Table S3. List of hydrogen bond interaction between 131-2a and postF (from PISA).

from residue	distance (Å)	to residue
	**F1-to-HC**	
E:LYS 390[NZ]	3.26	H:TYR 55[OH]
E:ASN 383[ND2]	3.76	H:TYR 59[OH]
E:LYS 390[NZ]	3.07	H:ASN 65[O]
E:ASP 385[OD1]	3.30	H:ASN 110[N]
E:ASP 385[OD2]	2.97	H:TYR 57[OH]
E:PRO 389[O]	2.86	H:TYR 55[OH]
	**F2-to-HC**	
B:TYR 33[OH]	2.87	H:TYR 59[OH]
B:SER 35[O]	2.98	H:ASN 110[ND2]
B:LYS 42 [NZ]	3.05	H:TYR 59[OH]
	**F1-to-LC**	
E:ASN 325[OD1]	2.99	L:ASN 66[ND2]
E:PHE 387[O]	3.73	L:TYR 116[OH]

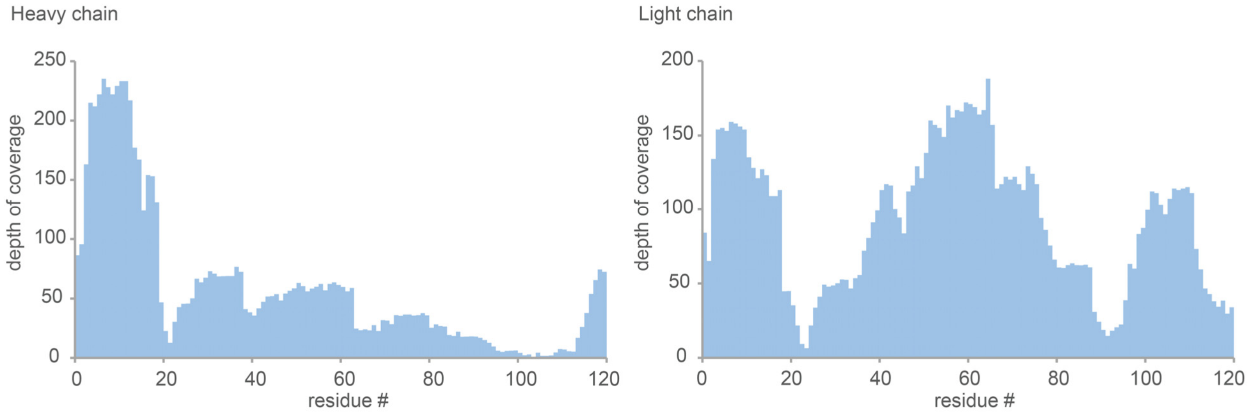

Supplementary Figure S1. Depth of coverage (total number of overlapping peptides mapped per position) for the variable domains of the 131-2a heavy and light chains.

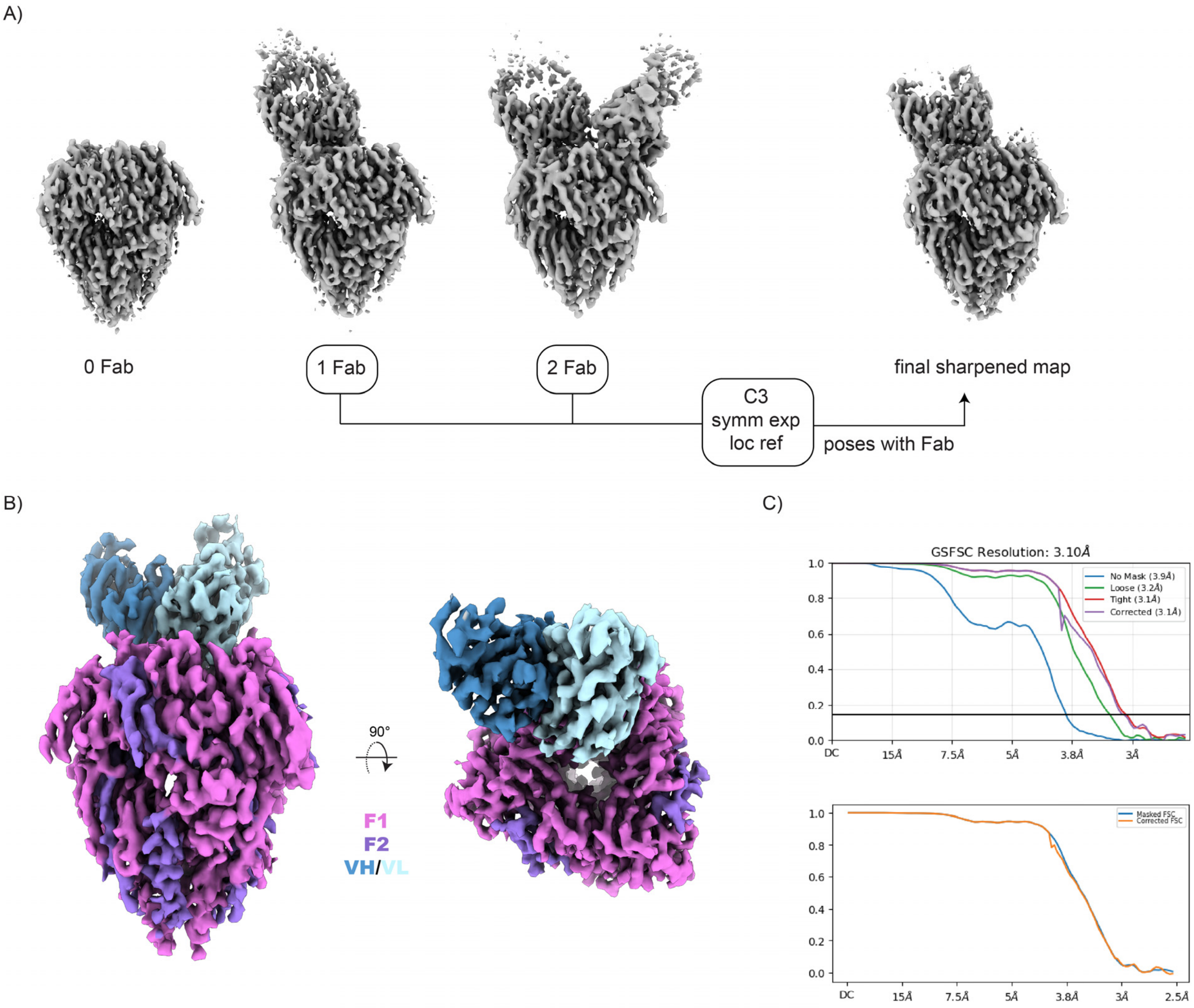

Figure S2. CryoEM single particle reconstruction of postF in complex with 131-2a Fab. A) Final local refinements of 3:0, 3:1 and 3:2 stoichiometries. Following C3 reconstruction with symmetry relaxation (maximization), the 3:1 and 3:2 maps were aligned, symmetry expanded, and the posed with bound Fabs combined in one local refinement to obtain the final map. B) Final sharpened map used for model building. C) FSC plot for the final local refinement (top) and sharpened map (bottom) used for model building.

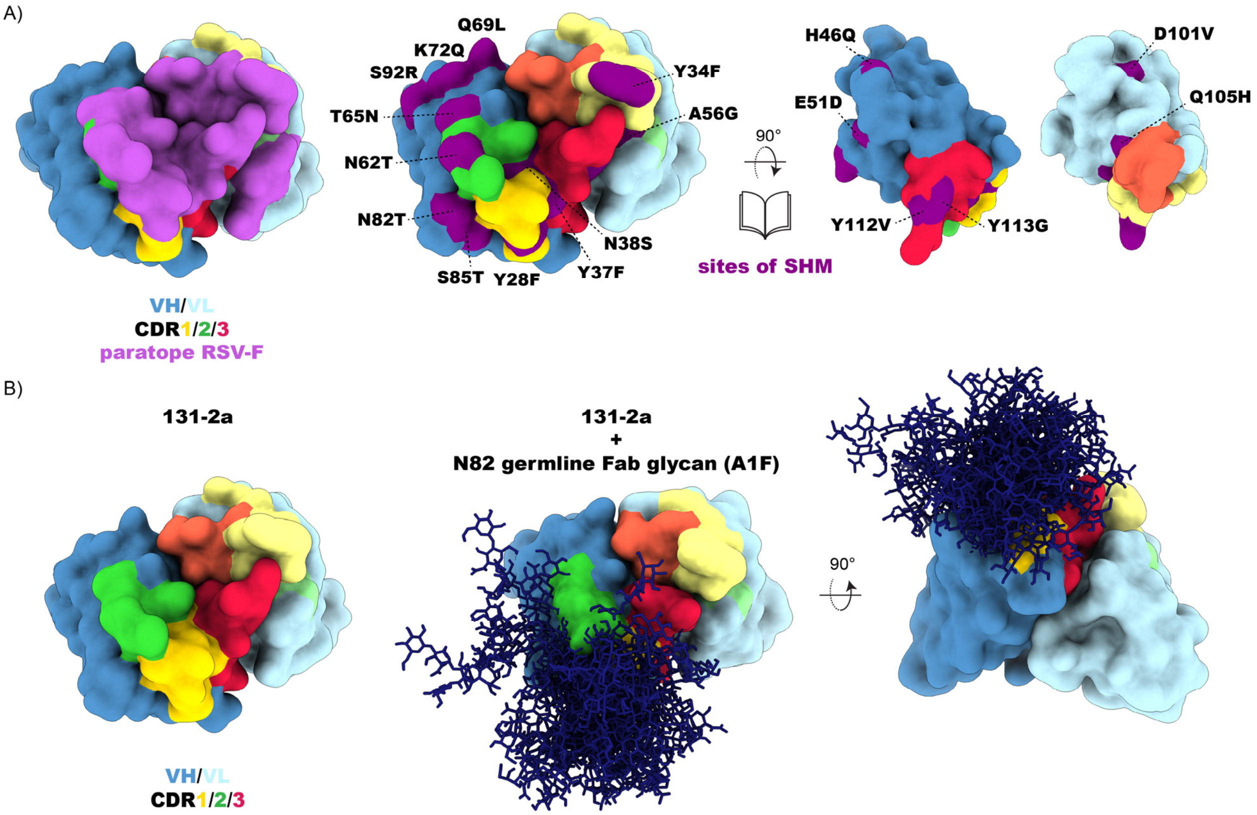

Figure S3. Analysis of inferred somatic hypermutation (SHM) in mature 131-2a sequence. A) Detailed view of paratope (left) compared to sites of SHM (middle), and VH-VL interface (right). B) Model of glycosylated 131-2a without the N82T mutation. An ensemble of 30 glycan conformation was grafted using GLYCOSHIELD, shown as dark blue sticks.

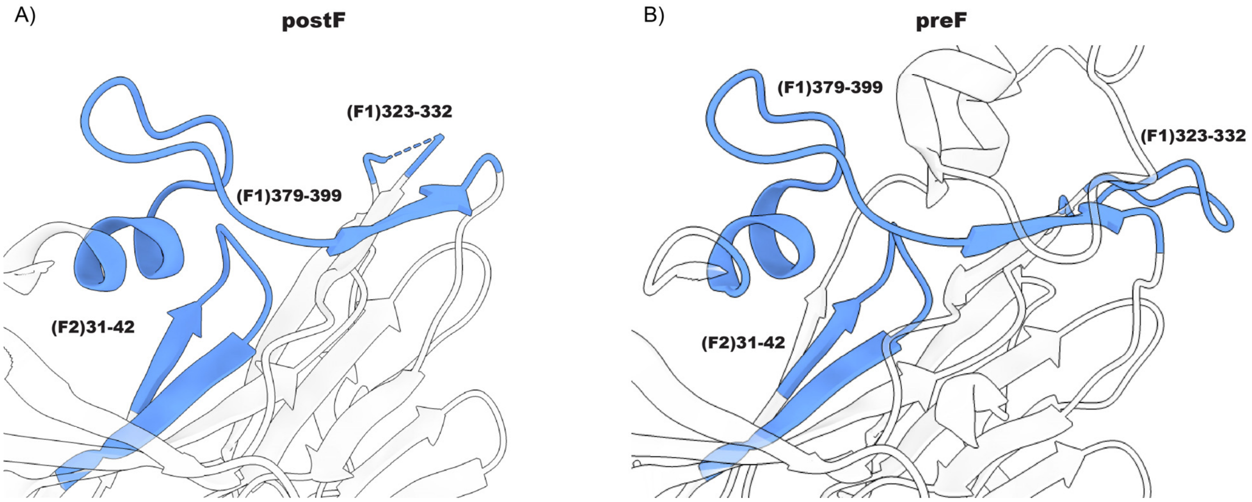

Supplementary Figure S4. Conformation of the 131-2a epitope in postF (A) and preF (B). The epitope is highlighted in blue color. Models used are PDB ID 3RRR and 4MMU for postF and preF, respectively.

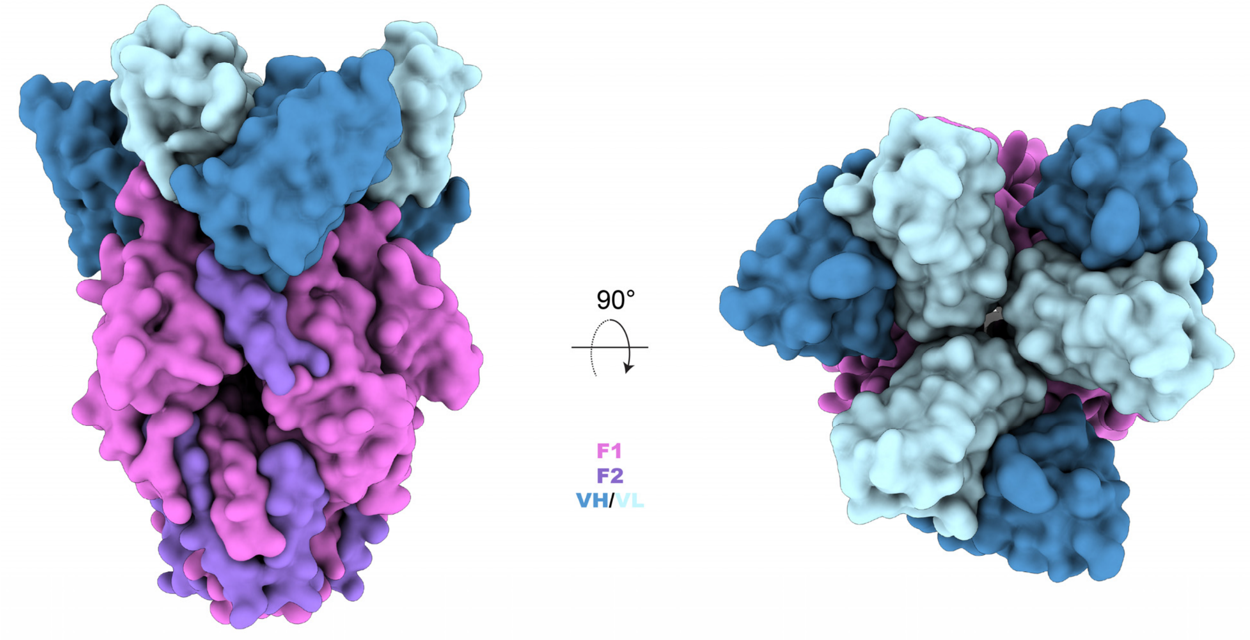

Supplementary Figure S5. Model of postF with 3 copies of 131-2a VH/VL bound. Model was generated by copying the resolved VH/VL domains over to the other protomers in the experimentally determined F:131-2a model. Note the lack of space between the light chains at the central cavity of the postF trimer.
